# Experiences of Social Withdrawal: Why Aging Matters Among Individuals With Hemophilia Facing Unexpected Longevity

**DOI:** 10.1093/geroni/igaa057.2312

**Published:** 2020-12-16

**Authors:** Tam Perry, Sara Schwartz, Dana Francis, Charles Kaplan

**Affiliations:** 1 Wayne State University, Detroit, Michigan, United States; 2 University of Southern California, Los Angeles, California, United States; 3 University of California San Francisco, San Francisco, California, United States

## Abstract

For the first time, individuals with hemophilia are living beyond their 30s and 40s. This cohort, many of whom had been dependent on donated blood and contracted HIV and hepatitis, face unique challenges as they age with hemophilia and other conditions. Little is known about the experiences and health needs of these adults over the age of 40. Scientific advances have changed the life course of individuals who received earlier treatment modalities. Today, a person with hemophilia who has received preventative synthetic treatment can look forward to a long, healthy and active life. Semi-structured telephone interviews (n=32) were conducted with long-term survivors, family members, and professionals who supported these families. Findings include shame, fear and coping through social withdrawal in order to hide their hemophilia to avoid assumptions of HIV status. Analyzing unexpected longevity and why age matters for this historically isolated cohort, we explore appropriate care models.

